# Physician recognition and documentation of sepsis. a comparison of the 2001 accp/sccm consensus conference definitions and physician documented diagnosis

**DOI:** 10.1186/2197-425X-3-S1-A224

**Published:** 2015-10-01

**Authors:** RJ Jolley, DW Yergens, H Quan, CJ Doig

**Affiliations:** University of Calgary, Community Health Sciences, Calgary, Canada; University of Calgary, Community Health Sciences, Calgary, Alberta Canada; O'Brien Institute of Public Health, Calgary, Canada; Department of Critical Care Medicine, University of Calgary, Calgary, Canada; Snyder Institute for Chronic Diseases, Calgary, Canada

## Intr

Estimates of sepsis incidence, cost of care and outcomes many times are derived from administrative health data. Translating a diagnosis of sepsis into administrative data involves health care coders reviewing the medical record and assigning diagnostic codes for each condition present. This coding and quality of the data are thus influenced by the physician's ability to both recognize and adequately document a diagnosis of sepsis, which can impact critical health care sector decisions.

## Objectives

To compare cases of sepsis, severe sepsis and septic shock that were identified through a reference standard medical record review using the 2001 ACCP/SCCM consensus criteria definitions [1] versus physician documented cases.

## Methods

Retrospective cohort study in which the medical records of ICU patients from three tertiary care centres in Calgary, Canada from years 2009-2012 were randomly selected and linked to an administrative discharge abstract database and ICU clinical database. Patient demographics and clinical information including a diagnosis of sepsis according to a checklist following the ACCP/SCCM 2001 consensus definition criteria, as well as whether the terms, 'sepsis', 'severe sepsis' or 'septic shock' were documented in the physician progress notes (physician 'explicit stated diagnosis) in the patient chart were collected. Summary statistics, frequencies and percentages were calculated.

## Results

Out of 945 ICU patient medical records reviewed, 583 patients (61.7%) were classified as having sepsis and 362 (38.3%) were classified as not having sepsis according to the 2001 ACCP/SCCM consensus definition. Of these 85 met criteria for sepsis, 195 for severe sepsis, and 303 met criteria for septic shock. According to the physician 'explicit' stated diagnosis, 382 out of 945 (40.42%) patients had one of the terms, 'sepsis', 'severe sepsis' or 'septic shock' documented in the chart. Multiple diagnosis terms were used in 52.3% of physician identified cases. Of the 583 cases identified by the consensus definition only 390 of these cases (64.6%) were documented by physicians (sepsis = 5.2%, severe sepsis = 23.8% and septic shock = 69.4%). In physician documented cases, 36.1% had mean SOFA scores >11, 63.9% had APACHE score>20 and 74.6% had a microbiologically confirmed infection.

## Conclusions

These results suggest that a diagnosis of sepsis is poorly documented in medical records which may be reflective of the ability of a physician to recognize less severe forms of sepsis. Factors affecting physician documentation and diagnosis must be further studied to understand the impact on data quality.

## Grant Acknowledgment

We acknowledge the Alberta Sepsis Network Grant through the Alberta Innovates: Health Solutions.
